# A comparative study on early prediction of venous thromboembolism in patients with traumatic brain injury by machine learning model

**DOI:** 10.1186/s12959-025-00772-2

**Published:** 2025-10-09

**Authors:** Chuntao Wang, Mengqi Chen, Kan Wang, Ling Pu, Siyuan Qi, Zhaofeng Kang, Wei Wang, Tao Liu, Weiming Xie, Xiangjun Bai, Zhanfei Li, Xijie Dong, Qiqi Wu

**Affiliations:** 1 Division of Trauma Surgery, Emergency Surgery & Surgical Critical, Tongji Trauma Center, Tongji Medical College, Huazhong University of Science and Technology, Wuhan, 430030 P.R. China; 2 Department of Emergency and Critical Care Medicine, Tongji Hospital, Tongji Medical College, Huazhong University of Science and Technology, Wuhan, 430030 China; 3 Department of Respiratory and Critical Care Medicine, Tongji Hospital, Tongji Medical College, Huazhong University of Science and Technology, Wuhan, Hubei 430030 China; 4 Department of Nursing, Tongji Hospital, Tongji Medical College, Huazhong University of Science and Technology, 1095 Jiefang Avenue, Wuhan, 430030 China; 5 School of Nursing, Tongji Medical College, Huazhong University of Science and Technology, 13 Hangkong Road, Wuhan, 430030 China; 6 Division of Nephrology, Tongji Medical College, The Fifth Hospital of Wuhan City, Huazhong University of Science and Technology, Wuhan, 430030 P.R. China; 7 Traumatic Surgery, Tongji Hospital, Tongji Medical College, Huazhong University of Science and Technology, Wuhan, 430030 P.R. China

**Keywords:** Traumatic brain injury, Venous thromboembolism, D-dimer, Prediction model, Machine learning

## Abstract

**Objective:**

We aimed to evaluate the predictive value of the post-injury D-dimer decrease rate for venous thromboembolism (VTE) in patients with traumatic brain injury (TBI). Additionally, we sought to establish a practical and efficient prediction model using a machine-learning algorithm to facilitate the early identification of high-risk individuals for VTE following TBI.

**Methods:**

This study encompassed patients over the age of 18 with TBI who were admitted to our trauma center, between May 2018 and December 2021. The participants were allocated into training (70%) and validation (30%) cohorts. Within the training cohort, predictive models were developed using the generalized linear model (GLM), least absolute shrinkage and selection operator model (LSM), and random forest model (RFM), based on the clinical characteristics of the patients. The predictive accuracy of these models was assessed through the area under the receiver operating characteristic curve (AUROC). The stability and clinical practicability of the models were evaluated using a calibration curve and a clinical impact curve. The repeatability and reliability of the models were confirmed through a validation dataset.

**Results:**

A total of 1,108 patients aged over 18 years with TBI who met the inclusion criteria were included in this study. Post-injury D-dimer on the third day (PDD3) and the post-injury D-dimer decreasing rate on the third day (PDDR3) were common predictors across the three models and were closely related to VTE for patients with TBI. The area under the receiver operating characteristic curve (AUROC) for the GLM, LSM, and RFM in the training cohort were 0.84 (95% confidence interval [CI]: 0.80–0.87), 0.85 (95% CI: 0.82–0.88), and 0.82 (95% CI: 0.78–0.86), respectively. In the verification cohort, the AUROC values were 0.85 (95% CI: 0.79–0.90), 0.85 (95% CI: 0.79–0.91), and 0.79 (95% CI: 0.73–0.86), respectively. The calibration curves of the three prediction models agree well with the actual observed results. All models showed a high clinical net income in the decision and clinical impact curves.

**Conclusion:**

PDD3 and PDDR3 emerged as effective indices for predicting VTE in patients with TBI. We formulated a practical predictive model based on PDDR3, demonstrating satisfactory performance in forecasting VTE, which will assist clinicians in the early identification and initiation of PTP treatment for TBI patients.

## Introduction

Traumatic brain injury (TBI) represents one of the most prevalent forms of traumatic injury, contributing to a rapidly escalating global disease burden [1]. Patients admitted to the hospital following TBI are at an elevated risk for venous thromboembolism (VTE), which encompasses conditions such as deep vein thrombosis and pulmonary embolism (PE) [2]. PE, in particular, is a severe complication associated with an estimated mortality rate of approximately 30% among TBI patients [3]. Post-thromboembolic syndromes, including phlebitis, valvular insufficiency, lower extremity swelling, lower limb pain, and venous ulcers, constitute a significant public health concern [4]. Consequently, the implementation of effective strategies to prevent VTE events following TBI is imperative.

Pharmacological thromboprophylaxis (PTP) is integral in reducing the risk of VTE following TBI. Nonetheless, the initiation of PTP in TBI patients is often delayed due to concerns regarding the potential for secondary hemorrhage [5]. Although certain studies indicate that early administration of appropriate PTP does not elevate the risk of secondary hemorrhagic events, the current body of evidence is insufficient to support a universal recommendation for early anticoagulation therapy in all TBI patients. Some scholars contend that premature anticoagulation may result in life-threatening secondary hemorrhagic complications [6]. Consequently, there is an urgent need to develop effective diagnostic methods to promptly and accurately identify individuals at high risk for VTE following TBI [7]. Several researchers have reported that high D-dimer levels after TBI may be a key predictor [8].

D-dimer levels are recognized for their high negative predictive value, although their positive predictive value remains relatively low [9]. D-dimer levels typically exhibit a rapid increase followed by a gradual return to baseline in some patients with TBI [10]. Clinical observations indicate that certain patients experience a slower decline or persistently elevated D-dimer levels, which are often associated with coagulation disorders. Despite this, the relationship between the post-injury D-dimer decreasing rate (PDDR) and TBI has not been extensively studied [11]. We propose that PDDR may serve as a significant predictor of VTE.

In recent years, efforts have been made to develop predictive models for VTE following trauma using traditional multiple regression analyses. However, the predictive accuracy of these models has been found to be suboptimal [12]. Currently, novel machine learning algorithms, which leverage clinical features, show considerable promise in various domains of medical research, particularly in the development and fitting of predictive models [13, 14]. Heo [15] et al. developed three machine learning models utilizing 38 retrospective clinical parameters from patients with acute ischemic stroke to distinguish high-risk individuals with poorer prognoses, achieving an Area Under Receiver Operating Characteristic curve (AUROC) of 0.888. Churpek [16] et al. constructed eight supervised machine learning models based on 29 clinical characteristics, with the random forest model demonstrating the best performance in distinguishing critically ill patients in wards, achieving an area under curve (AUC) value of 0.80. Although machine learning algorithms have demonstrated significant advantages in constructing clinical prediction models compared to traditional methods, only Peng [17] et al. have attempted to develop prediction models using machine learning algorithms for complications associated with trauma patients during hospitalization. The model they constructed greatly improved prediction efficiency compared to conventional methods. Consequently, our study aimed to assess the predictive value of PDDR for the risk of VTE in patients with TBI. Additionally, we sought to develop a simple and efficient clinical model for predicting VTE using machine learning techniques.

## Methods

### Study population

**Fig. 1:**
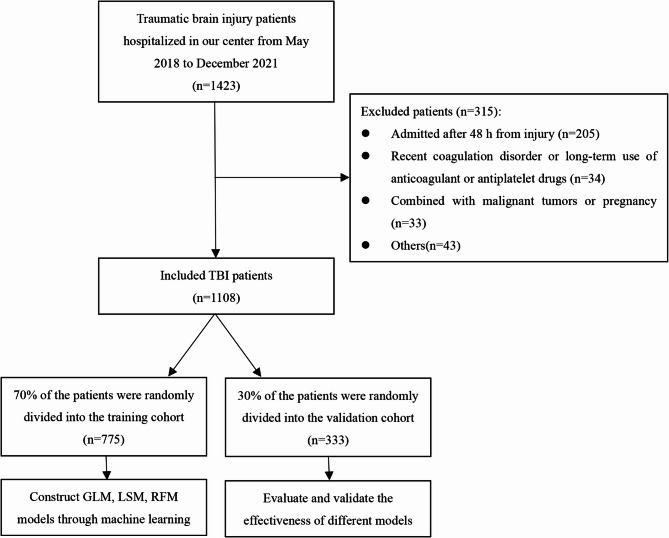
Flow chart of patient selection and study design

We performed a retrospective analysis of all patients admitted to our trauma center from May 1, 2018, to December 31, 2021, who were diagnosed with TBI. The exclusion criteria were as follows: (1) pregnant or underage patients; (2) patients not admitted on the day following injury; (3) recent coagulation insufficiency or use of anticoagulant or antiplatelet medications; (4) malignant neoplastic disease; (5) combined with active bleeding from other parts of the body other than the head; (6) incomplete data. Finally, a total of 1,108 patients with TBI who met the inclusion criteria were included in our study. The flow chart of patient selection and study design is shown in Fig. 1. This study was approved by the Medical Ethics Committee of Tongji Medical College, Huazhong University of Science and Technology.

### Data collection

Comprehensive clinical data were collected, encompassing demographic characteristics, trauma-related information, and various clinical indicators. Demographic data included age, gender, body mass index (BMI), smoking history, and medical history of pre-existing conditions. Trauma-related information comprised the mechanism of injury, the injury severity score (ISS), abbreviated injury scale (AIS), Glasgow coma scale (GCS), and injury sites. Clinical indicators encompassed blood biochemical markers and other relevant clinical indicators. The blood biochemical indices include white blood cell count, hemoglobin, platelets, prothrombin time (PT), international normalized ratio (INR), fibrinogen (FIB), activated partial thromboplastin time (APTT), and estimated glomerular filtration rate (eGFR). Post-injury D-dimer on the first day (PDD1), post-injury D-dimer on the third day (PDD3), and post-injury D-dimer decrease rate on the third day (PDDR3 = PDD3/PDD1) were also calculated and recorded. Additional clinical indicators included surgery, lower extremity (LE) surgery, craniotomy, thoracotomy, laparotomy, LE fractures, ventilation, transfusion on the first day, PTP, physical thromboprophylaxis, and severe head injury (head AIS score > 3). Descriptive statistical analyses were conducted for all variables.

### Study method

The diagnosis of TBI was established based on the patient’s injury history, clinical presentation, and radiological assessment. VTE diagnoses were initially determined via ultrasonography and subsequently confirmed by an attending radiologist. In cases where the diagnosis was uncertain, computed tomography angiography (CTA) was employed as an additional diagnostic tool. For the diagnosis of pulmonary embolism, CTA of the pulmonary artery was utilized to provide a definitive diagnosis for all patients.

### Model development and evaluation

The research cohort was randomly divided into two groups: 70% of the participants were allocated to the training cohort, while the remaining 30% were assigned to the validation cohort. The training cohort was used for model development, and the validation cohort was employed for internal validation to assess the model’s validity and reproducibility. We employed generalized linear model (GLM), adaptive least absolute shrinkage and selection operator (LASSO) regression, and random forest (RF) methodologies to construct predictive models for VTE events in patients with TBI. In this study, a stepwise approach was utilized in the multivariate logistic regression analysis to identify significant variables for developing a generalized linear model (GLM). The LASSO is a well-established feature selection technique that effectively isolates meaningful features from a large set of potentially multicollinear variables, facilitating the construction of the LASSO [18] model (LSM). The RF method comprises numerous independent decision trees that function collectively. These diverse decision tree models were employed in the development of the random forest [19] model (RFM). The significance of candidate variables was assessed using the mean decrease in the Gini coefficient (Mean Decrease Gini, MDG).

The model’s predictive performance, stability, and validity were evaluated using the receiver operating characteristic curve (ROC) and calibration curve. Decision curve analysis (DCA) and clinical impact curve (CIC) were utilized to assess the clinical utility of the models. The clinical applicability of each model was determined through DCA and CIC using the “rms” and “rmda” packages, while the area under the curve (AUC) was calculated using the “rms” package.

### Statistical analysis

Continuous variables were first analyzed using the Shapiro-Wilk test to assess normality. Data that were normally distributed were expressed as mean ± standard deviation and analyzed by the independent samples t-test; non-normally distributed data were expressed as median (interquartile range) and analyzed by the Mann-Whitney U test. Categorical data were described as percentages and analyzed by the chi-square test. All *p* values are two-sided, and differences were considered statistically significant at *p* < 0.05. Statistical analyses were performed using IBM SPSS Statistics for Windows (version 26; IBM Corp, Armonk, NY) and R Statistical Software (version 4.1.2, https://www.r-project.org).

## Results

### Comparison of the training and validation cohorts

The participants were randomly assigned to either the training cohort (*n* = 775, 70%) or the validation cohort (*n* = 333, 30%) as illustrated in Fig. 1. A comprehensive summary of the clinical characteristics for both cohorts is provided in Table 1. Within the overall cohort, 182 individuals (16.4%) were diagnosed with VTE, compared to 130 (16.8%) in the training cohort and 52 (15.6%) in the validation cohort. No statistically significant differences were identified between the cohorts (*p* > 0.05). Similarly, other indicators also did not exhibit statistically significant differences between the training and validation cohorts (*p* > 0.05), suggesting that the baseline characteristics of the cohorts are comparable.

**Table 1:** Comparison of characteristics between the training and validation cohort

Characteristics	Validation cohort (*N* = 333)	Training cohort (*N* = 775)	*P*value
VTE, n (%)	52(15.6%)	130(16.8%)	0.633
Male, n (%)	216(64.9%)	511(65.9%)	0.731
Age, n (%)	49.2 ± 15.3	49.3 ± 15.0	0.860
Smoking, n (%)	54(16.2%)	123(15.9%)	0.886
Hypertension, n (%)	67(20.1%)	161(20.8%)	0.805
Diabetes, n (%)	28(8.4%)	58(7.5%)	0.598
Hematological Disease, n (%)	19(5.7%)	35(4.5%)	0.400
BMI > 25, n (%)	48(14.4%)	110(14.2%)	0.923
ISS	17.0 ± 10.2	18.7 ± 10.8	0.489
GCS	12.8 ± 3.5	12.1 ± 4.0	0.118
Spinal fracture, n (%)	89(26.7%)	202(26.1%)	0.818
Transfusion in the First day, n (%)	60(18%)	157(20.3%)	0.389
Surgery, n (%)	105(31.5%)	278(35.9%)	0.164
Craniotomy, n (%)	30(9%)	82(10.6%)	0.427
Thoracotomy, n (%)	8(2.4%)	14(1.8%)	0.516
Laparotomy, n (%)	18(5.4%)	66(8.5%)	0.075
LE surgery, n (%)	73(21.9%)	163(21%)	0.740
Chemoprophylaxis, n (%)	36 (10.8%)	85 (11%)	0.939
Physical prophylaxis, n (%)	177(53.2%)	416(53.7%)	0.873
Vasoactive agents, n (%)	29(8.7%)	88(11.4%)	0.190
Antiarrhythmic drugs, n (%)	9(2.7%)	12(1.5%)	0.202
Sedative, n (%)	72(21.6%)	172(22.2%)	0.833
Hemostatic, n (%)	100(30%)	253(32.6%)	0.392
Emergency operation, n (%)	50(15%)	98(12.6%)	0.288
Ventilation, n (%)	44(13.2%)	114(14.7%)	0.514
Pneumonia, n (%)	38(11.4%)	107(13.8%)	0.279
Sepsis, n (%)	13(3.9%)	50(6.5%)	0.097
Shock, n (%)	41(12.3%)	95(12.3%)	0.980
CVC, n (%)	79(23.7%)	212(27.4%)	0.208
WBC(×10^9^/L)	8.91 [6.91;12.1]	9.20 [7.18;12.1]	0.259
HB(g/L)	115 [100;131]	112 [95.0;128]	0.134
Plt(×10^9^/L)	164 [123;208]	159 [117;202]	0.285
PT(s)	14.3 [13.7;15.2]	14.3 [13.7;15.3]	0.309
APTT(s)	37.0 [34.2;40.2]	37.0 [34.3;41.0]	0.090
INR	1.11 [1.05;1.20]	1.11 [1.05;1.21]	0.346
FIB(g/L)	2.93 [2.35;3.74]	2.93 [2.33;3.74]	0.779
PDD1(µg/mL)	7.55 [3.29;15.9]	8.38 [3.41;17.9]	0.570
PDDR3(µg/mL)	0.52 [0.30;0.79]	0.53 [0.33;0.78]	0.537
TC (mmol/L)	3.57 [2.98;4.11]	3.45 [2.82;4.11]	0.204
TG (mmol/L)	0.90 [0.59;1.30]	0.90 [0.60;1.36]	0.136
HDL (mmol/L)	1.03 [0.82;1.23]	1.03 [0.79;1.23]	0.233
LDL (mmol/L)	2.11 [1.51;2.83]	2.10 [1.62;2.55]	0.216
eGFR(mL/min)	88.6 [79.1;94.5]	88.5 [75.9;93.7]	0.550
Head AIS > 3, n (%)	61(18.3%)	165(21.3%)	0.261
LE fracture, n (%)	189(56.8%)	430(55.5%)	0.696
PDD3	2.97 [1.24;8.39]	3.88 [1.56;9.31]	0.552

*VTE* Venous thromboembolism, *ICU* Intensive unit care, *BMI* Body mass index, *ISS* Injury severity score, *GCS* Glasgow coma scale, *CVC* Central venous catheter, *WBC* White blood cells, *HB* Hemoglobin, *Plt* Blood platelet, *PT* Prothrombin time, *APTT* Activated partial thromboplastin time, *INR* International normalized ratio, *FIB* Fibrinogen, *PDD1* Post-injury D-dimer on the first day, *PDD3* Post-injury D-dimer on the third day, *PDDR3* Post-injury D-dimer decrease rate on the third day, *TC* Total cholesterol, *TG* Triglyceride, *HDL* High density lipoprotein, *LDL* Low density lipoprotein, *eGFR* Estimated glomerular filtration rate, *AIS* of the most prevalent forms of traumatic injury, contributin eviated injury scale, *LE* Lower extremity

### Logistic regression analysis

**Fig. 2:**
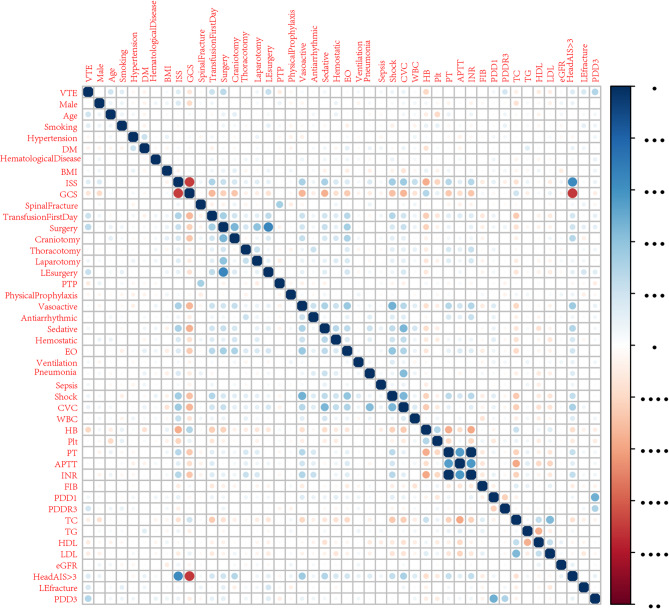
Correlation matrix between candidate variables. The size and color of the circle in the matrix reflect the correlation between the corresponding variables. The darker the blue, the stronger the positive correlation between variables, and the darker the red, the stronger the negative correlation between variables. VTE: Venous thromboembolism; ICU: Intensive unit care; BMI: Body mass index; ISS: Injury severity score; GCS: Glasgow coma scale; CVC: Central venous catheter; WBC: White blood cells; HB: Hemoglobin; Plt: Blood platelet; PT: Prothrombin time; APTT: Activated partial thromboplastin time; INR: International normalized ratio; FIB: Fibrinogen; PDD1: Post-injury D-dimer on the first day; PDD3: Post-injury D-dimer on the third day; PDDR3: Post-injury D-dimer decrease rate on the third day; TC: Total cholesterol; TG: Triglyceride; HDL: High density lipoprotein; LDL: Low density lipoprotein; eGFR: Estimated glomerular filtration rate; AIS: Abbreviated injury scale; LE: Lower extremity

The findings from the univariate and multivariate logistic regression analyses conducted on the training cohort are presented in Table 2. The univariate analysis identified 16 variables that were significantly associated with VTE in patients with TBI. These factors included age (OR: 1.04, 95% confidence interval (CI): 1.02–1.05, *p* < 0.001), smoking (OR: 2.21, 95% CI: 1.41–3.47, *p* < 0.001), ISS (OR: 1.03, 95% CI: 1.01–1.05, *p* < 0.001), GCS (OR: 0.92, 95% CI: 0.88–0.96, *p* < 0.001), transfusion in the first day (OR: 3.28, 95% CI: 2.18–4.94, *p* < 0.001), surgery (OR: 4.25, 95% CI: 2.85–6.31, *p* < 0.001), LE surgery (OR: 3.49, 95% CI: 2.33–5.24, *p* < 0.001), pharmacological thromboprophylaxis (OR: 9.20, 95% CI: 6.06–13.98, *p* < 0.001), shock (OR: 1.71, 95% CI: 1.02–2.86, *p* = 0.040), central venous catheter (OR: 1.65, 95% CI: 1.11–2.46, *p* = 0.014), HB (OR: 0.98, 95% CI: 0.97–0.99, *p* < 0.001), TC (OR: 0.79, 95% CI: 0.66–0.96, *p* = 0.016), LDL (OR: 0.58, 95% CI: 0.45–0.77, *p* < 0.001), HDL (OR: 0.43, 95% CI: 0.25–0.76 (*p* = 0.004)), head AIS > 3 (OR: 2.32, 95% CI: 1.53–3.50, *p* < 0.001), lower extremity fracture (OR: 2.63, 95% CI: 1.73-4.00, *p* < 0.001), PDD3 (OR: 1.07, 95% CI: 1.05–1.09, *p* < 0.001), and PDDR3 (OR: 1.43, 95% CI: 1.23–1.67, *p* < 0.001). Notably, the results indicate that PDD3 and PDDR3 may serve as potential predictors of VTE. The correlation analysis of the candidate variables is depicted in Fig. 2.

**Table 2:** Univariate and multivariate logistic regression analysis of risk factors for VTE in patients with TBI in training cohort

Characteristics	Control group (*N* = 645)	VTE group (*N* = 130)	univariate analysis				multivariate analysis
			OR (95%CI, *p* value)				OR (95%CI, *p* value)
Male, n (%)	423(65.6%)	88(67.7%)	1.10(0.74–1.64, *p* = 0.643)				
Age, n (%)	48.0 ± 15.3	55.9 ± 11.7	1.04(1.02–1.05, *p* < 0.001)				1.04(1.03–1.06, *p* < 0.001)
Smoking, n (%)	89(13.8%)	34(26.2%)	2.21(1.41–3.47, *p* < 0.001)				1.95 (1.11–3.40, *p* = 0.019)
Hypertension, n (%)	139(21.6%)	22(16.9%)	0.74(0.45–1.22, *p* = 0.237)				
Diabetes, n (%)	53(8.2%)	5(3.8%)	0.45(0.18–1.14, *p* = 0.092)				
Hematological Disease, n (%)	28(4.3%)	7(5.4%)	1.25(0.54–2.94, *p* = 0.602)				
BMI > 25, n (%)	88(13.6%)	22(16.9%)	1.29(0.77–2.15, *p* = 0.329)				
ISS	18.0 ± 10.9	21.8 ± 9.2	1.03(1.01–1.05, *p* < 0.001)				0.96(0.92–1.01, *p* = 0.094)
GCS	12.3 ± 3.9	10.9 ± 4.4	0.92(0.88–0.96, *p* < 0.001)				0.98(0.87–1.10, *p* = 0.703)
Spinal fracture, n (%)	171(26.5%)	31(23.8%)	0.87(0.56–1.35, *p* = 0.528)				
Transfusion in the First day, n (%)	106(16.4%)	51(39.2%)	3.28(2.18–4.94, *p* < 0.001)				2.19 (1.26–3.80, *p* = 0.005)
Surgery, n (%)	194(30.1%)	84(64.6%)	4.25(2.85–6.31, *p* < 0.001)				2.37 (1.29–4.37, *p* = 0.006)
Craniotomy, n (%)	68(10.5%)	14(10.8%)	1.02(0.56–1.88, *p* = 0.939)				
Thoracotomy, n (%)	11(1.7%)	3(2.3%)	1.36(0.37–4.95, *p* = 0.639)				
Laparotomy, n (%)	50(7.8%)	16(12.3%)	1.67(0.92–3.04, *p* = 0.092)				
LE surgery, n (%)	109(16.9%)	54(41.5%)	3.49(2.33–5.24, *p* < 0.001)				1.04 (0.56–1.95, *p* = 0.902)
Chemoprophylaxis, n (%)	81(12.6%)	74(56.9%)	9.20(6.06–13.98, *p* < 0.001)				0.33 (0.11–0.97, *p* = 0.043)
Physical prophylaxis, n (%)	352(54.6%)	64(49.2%)		0.81(0.55–1.18, *p* = 0.266)			
Vasoactive agents, n (%)	70(10.9%)	18(13.8%)		1.32(0.76–2.30, *p* = 0.328)			
Antiarrhythmic drugs, n (%)	9(1.4%)	3(2.3%)		1.67(0.45–6.25, *p* = 0.447)			
Sedative, n (%)	136(21.1%)	36(27.7%)		1.43(0.93–2.20, *p* = 0.099)			
Hemostatic, n (%)	209(32.4%)	44(33.8%)		1.07(0.72–1.59, *p* = 0.749)			
Emergency operation, n (%)	83(12.9%)	15(11.5%)		0.88(0.49–1.59, *p* = 0.677)			
Ventilation, n (%)	90(14%)	24(18.5%)		1.40(0.85–2.29, *p* = 0.187)			
Pneumonia, n (%)	87(13.5%)	20(15.4%)		1.17(0.69–1.98, *p* = 0.568)			
Sepsis, n (%)	38(5.9%)	12(9.2%)		1.62(0.82–3.20, *p* = 0.161)			
Shock, n (%)	72(11.2%)	23(17.7%)		1.71(1.02–2.86, *p* = 0.040)		0.88 (0.43–1.83, *p* = 0.742)	
CVC, n (%)	165(25.6%)	47(36.2%)		1.65(1.11–2.46, *p* = 0.014)		0.89 (0.51–1.54, *p* = 0.667)	
WBC(×109/L)	9.12 [7.06;12.1]	9.34 [7.42;12.1]		1.01(0.97–1.06, *p* = 0.486)			
HB(g/L)	115 [100;131]	98.0 [84.2;110]		0.98(0.97–0.99, *p* < 0.001)		0.99 (0.98-1.00, *p* = 0.005)	
Plt(×109/L)	163 [123;202]	150 [106;202]		1.00(1.00–1.00, *p* = 0.732)			
PT(s)	14.3 [13.7;15.2]	14.6 [14.0;15.9]		1.00(0.97–1.03, *p* = 0.960)			
APTT(s)	37.0 [34.3;40.4]	37.3 [34.7;42.0]		1.00(0.99–1.01, *p* = 0.705)			
INR	1.11 [1.05;1.20]	1.14 [1.09;1.28]		0.98(0.72–1.34, *p* = 0.920)			
FIB(g/L)	2.93 [2.38;3.72]	2.95 [1.98;3.91]		1.00(0.93–1.08, *p* = 0.974)			
PDD1(µg/mL)	7.01 [2.93;17.5]	13.4 [6.47;18.8]		1.01(1.00-1.02, *p* = 0.102)			
PDDR3(µg/mL)	0.49 [0.30;0.68]	0.83 [0.63;1.45]		1.43(1.23–1.67, *p* < 0.001)		1.25 (1.03–1.51, *p* = 0.021)	
TC (mmol/L)	3.55 [2.86;4.17]	3.12 [2.47;3.78]		0.79(0.66–0.96, *p* = 0.016)		1.10 (0.91–1.35, *p* = 0.332)	
TG (mmol/L)	0.88 [0.59;1.32]	1.02 [0.71;1.45]		1.02(0.85–1.22, *p* = 0.843)			
HDL (mmol/L)	1.05 [0.83;1.23]	0.84 [0.70;1.08]		0.43(0.25–0.76, *p* = 0.004)		0.50 (0.26–0.97, *p* = 0.04)	
LDL (mmol/L)	2.14 [1.64;2.62]	1.84 [1.49;2.51]		0.58(0.45–0.77, *p* < 0.001)		0.83 (0.60–1.16, *p* = 0.281)	
eGFR(mL/min)	88.6 [75.9;93.4]	86.7 [77.4;95.1]		1.00(0.99–1.02, *p* = 0.413)			
Head AIS > 3, n (%)	120(18.6%)	45(34.6%)		2.32(1.53–3.50, *p* < 0.001)		1.81 (0.82–3.96, *p* = 0.139)	
LE fracture, n (%)	334(51.8%)	96(73.8%)		2.63(1.73-4.00, *p* < 0.001)		2.46 (1.49–4.09, *p* < 0.001)	
PDD3	5.8 ± 8.5	12.8 ± 9.7		1.07(1.05–1.09, *p* < 0.001)		1.06 (1.04–1.08, *p* < 0.001)	

*ICU* Intensive unit care, *BMI* Body mass index, *ISS* Injury severity score, *GCS* Glasgow coma scale, *CVC* Central venous catheter, *WBC* White blood cells, *HB* Hemoglobin, *Plt* Blood platelet, *PT* Prothrombin time, *APTT* Activated partial thromboplastin time, *INR* International normalized ratio, *FIB* Fibrinogen, *PDD1* Post-injury D-dimer on the first day, *PDD3* Post-injury D-dimer on the third day, *PDDR3* Post-injury D-dimer decrease rate on the third day, *TC* Total cholesterol, *TG* Triglyceride, *HDL* High density lipoprotein, *LDL* Low density lipoprotein, *eGFR* Estimated glomerular filtration rate, *AIS* Abbreviated injury scale, *LE* Lower extremity

After adjusting for confound factors in the multivariate logistic model, it was determined that age, smoking status, transfusion on the first day, surgical intervention, PTP, HB, HDL, head AIS > 3, LE fracture, PDD3, and PDDR3 were significant predictors of VTE in TBI patients, suggesting that PDD3 and PDDR3 serve as independent risk factors for the development of VTE.

### Establishment of prediction models

**Fig. 3:**
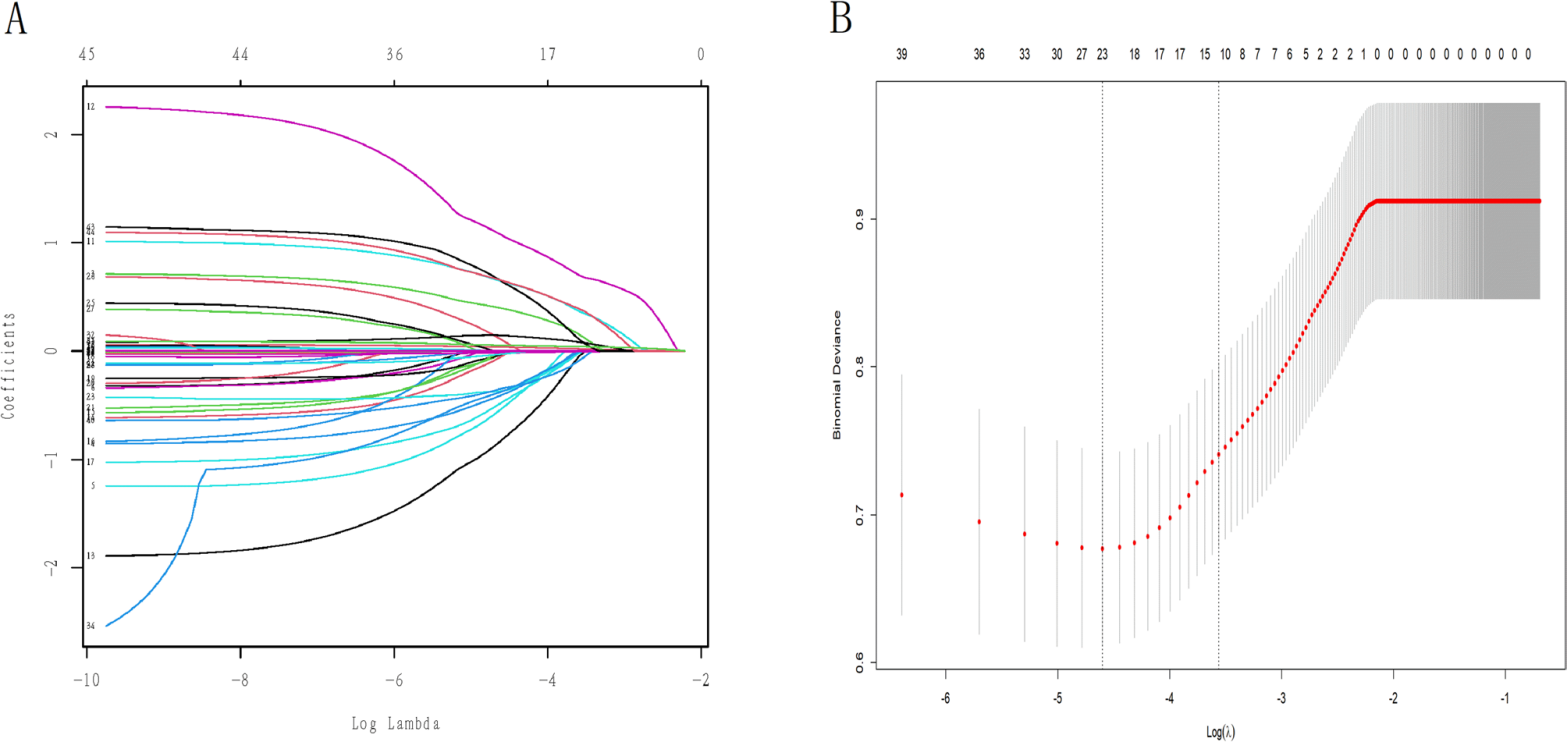
LSM feature screening. **A**: minimum absolute contraction of 45 features and selection of operator variable trajectory contours. Cross-validation with 50% discount; **B**: mean square error (MSE) diagrams of models under different lambda

**Fig. 4:**
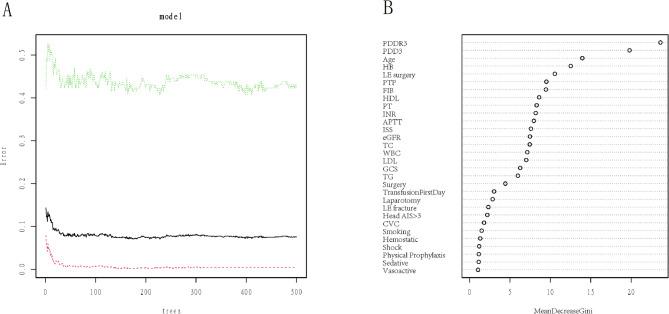
RFM model variable filtering. **A**: the relationship between the error and the number of random trees. There are three lines, green represents the error of the positive event group, red represents the error of the negative event group, and black represents the error of the total sample group; **B**: importance score of candidate features. PDD3: Post-injury D-dimer on the third day; PDDR3: Post-injury D-dimer decrease rate on the third day; HB: Hemoglobin; LE: Lower extremity; PTP: Pharmacological thromboprophylaxis; PT: Prothrombin time; APTT: Activated partial thromboplastin time; INR: International normalized ratio; FIB: Fibrinogen; TC: Total cholesterol; TG: Triglyceride; HDL: High density lipoprotein; LDL: Low density lipoprotein; eGFR: Estimated glomerular filtration rate; WBC: White blood cells; ISS: Injury severity score; GCS: Glasgow coma scale; CVC: Central venous catheter

As shown in Table 2, eleven variables were selected for the construction of the GLM model: age, smoking status, transfusion on the first day, surgical intervention, PTP, HB, HDL, head AIS > 3, LE fracture, PDD3, and PDDR3. A 5-fold cross-validation technique was employed. The optimal LSM was achieved when the initial set of 45 candidate variables was reduced to 10 using the LASSO method, as depicted in Fig. 3A and B. These selected variables included age, smoking status, transfusion on the first day, surgical intervention, PTP, HB levels, head AIS > 3, LE fracture, PDD3, and PDDR3. In RF model, the minimum error rate of 8.26% was observed for the entire sample group when the number of random trees was set to 166, as shown in Fig. 4A. The importance scores of the candidate variables are presented in Fig. 4B. Ultimately, six variables with high Mean Decrease Gini (MDG) scores were chosen to construct the RFM, comprising age, HB levels, PTP, LE fracture, PDD3, and PDDR3.

### Evaluation and validation of model performance

**Fig. 5:**
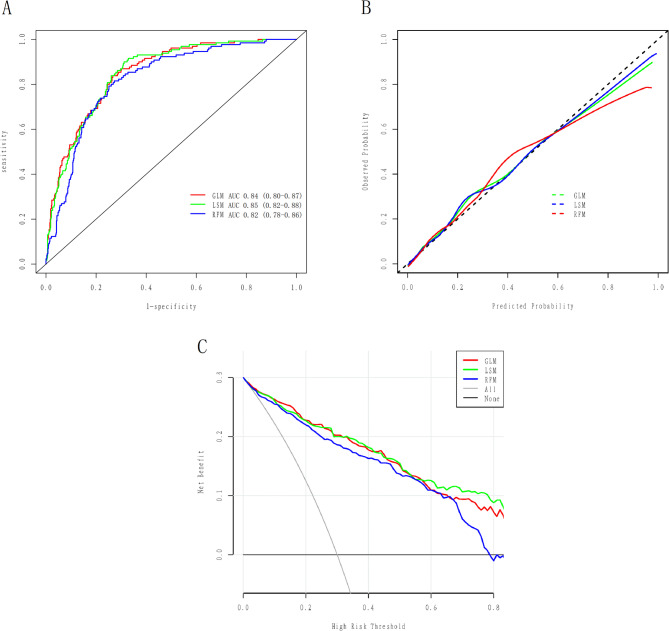
The effectiveness of VTE prediction models were evaluated in the training cohort. **A**: the subject working characteristic curve of the prediction models; **B**: the calibration curve of the prediction models; **C**: the decision curve analysis of the prediction models. GLM: Generalized linear model; LSM: Least absolute shrinkage and selection operator model; RFM: Random forest model; AUC: Area under the receiver operating characteristic curve

**Fig. 6:**
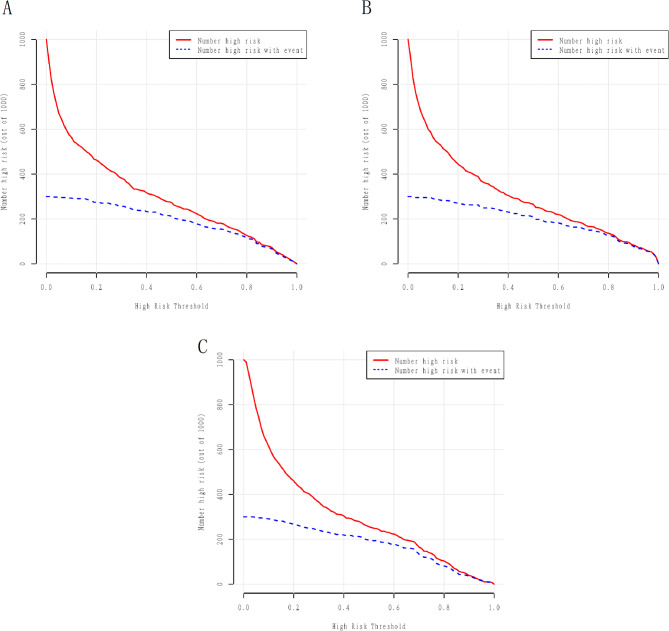
The clinical influence curves of the prediction models in the training cohort. **A**: clinical influence curve of generalized linear model (GLM); **B**: clinical influence curve of minimum absolute contraction and selection operator model (LSM); **C**: clinical influence curve of random forest model (RFM)

**Fig. 7:**
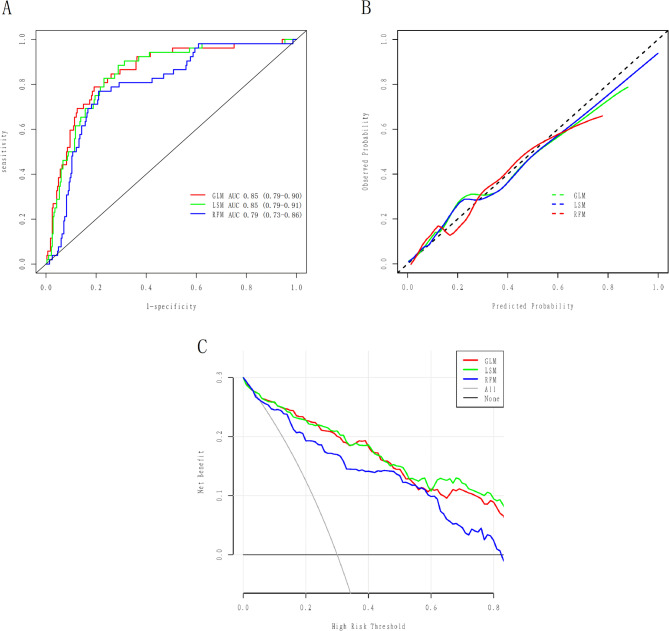
The effectiveness of VTE prediction models were evaluated in the validation cohort. **A**: the subject working characteristic curve of the prediction models; **B**: the calibration curve of the prediction models; **C**: the decision curve analysis of the prediction models. GLM: Generalized linear model; LSM: Least absolute shrinkage and selection operator model; RFM: Random forest model; AUC: Area under the receiver operating characteristic curve

**Fig. 8:**
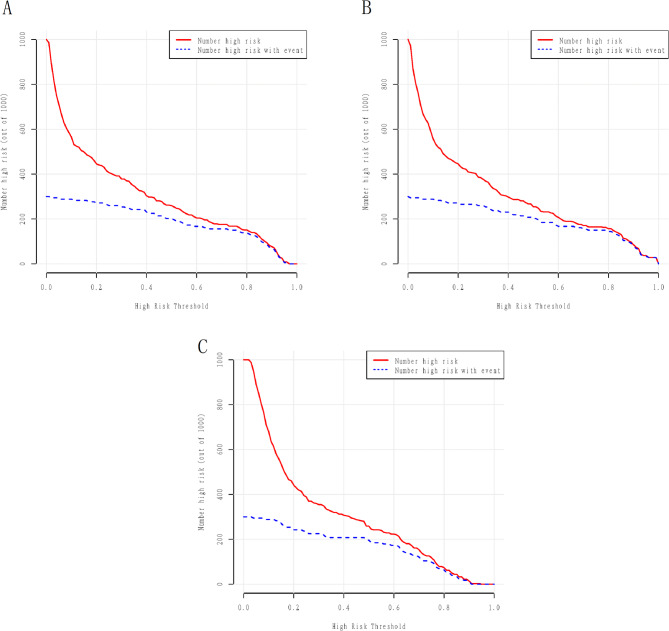
The clinical influence curves of the prediction models in the validation cohort. **A**: clinical influence curve of generalized linear model (GLM); **B**: clinical influence curve of minimum absolute contraction and selection operator model (LSM); **C**: clinical influence curve of random forest model (RFM)

Figure 5A demonstrated the ROC curves for GLM, LSM and RFM in the training cohort with AUCs of 0.84 (95% CI: 0.80–0.87), 0.85 (95% CI: 0.82–0.88), and 0.82 (95% CI: 0.78–0.86), respectively. All models exhibited satisfactory calibration in the training cohort (Fig. 5B). DCA and CIC showed that all three models conferred high net clinical benefit in the training cohort (Figs. 5C and 6). The ROC curves for all models in the validation cohort are shown in Fig. 7A, with AUCs of 0.85 (95% CI: 0.79–0.90), 0.85 (95% CI: 0.79–0.91), and 0.79 (95% CI: 0.73–0.86), respectively. All models showed satisfactory calibration and clinical applicability (Figs. 7B and C and 8).

### Evaluation of the importance of predictors

**Fig. 9:**
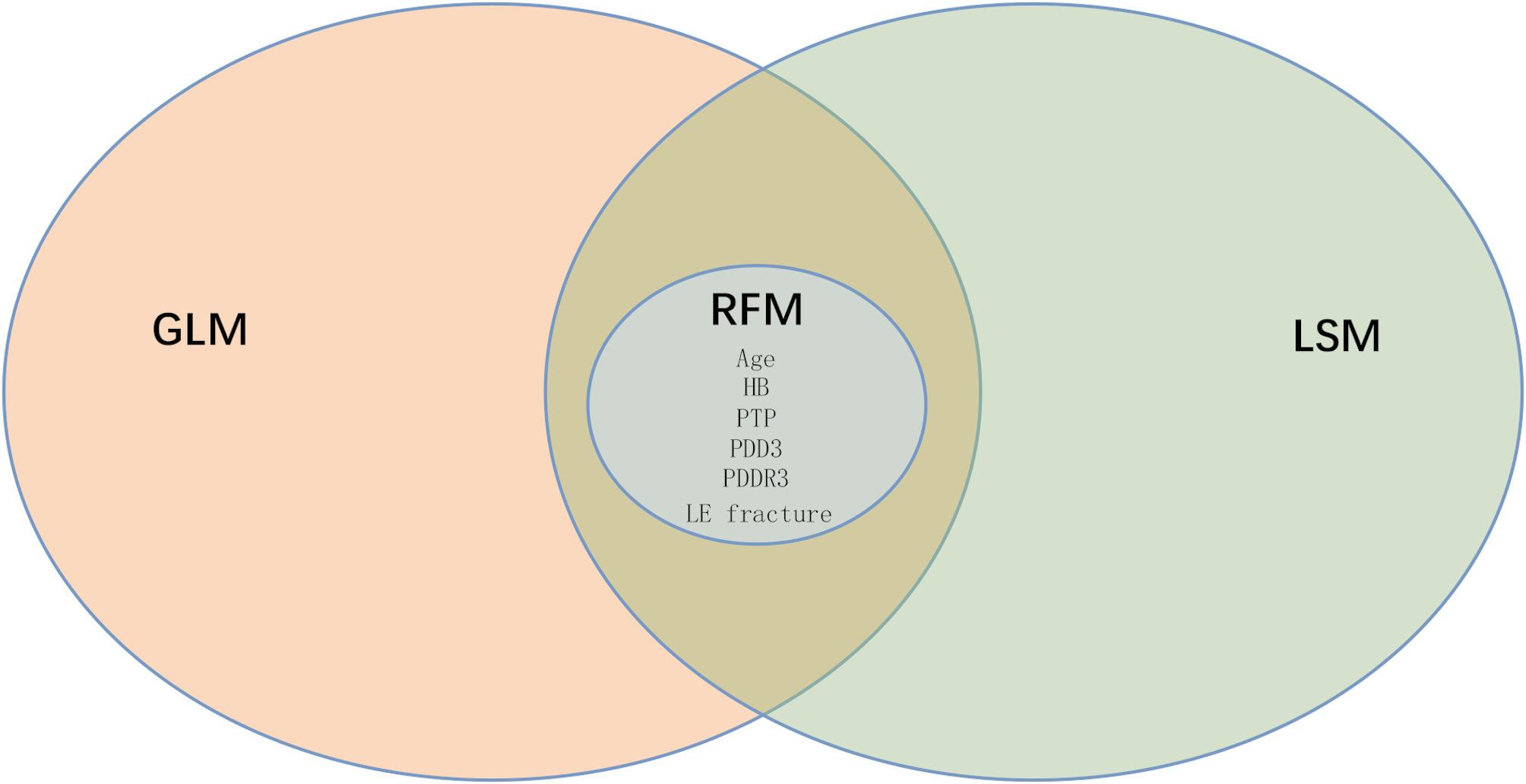
Cross-sectional variables between generalized linear model (GLM), LASSO model (LSM) and random forest model (RFM). GLM: Generalized linear model; LSM: Least absolute shrinkage and selection operator model; RFM: Random forest model; PDD1: Post-injury D-dimer on the first day; PDD3: Post-injury D-dimer on the third day; PDDR3: Post-injury D-dimer decrease rate on the third day; HB: Hemoglobin; PTP: Pharmacological thromboprophylaxis; LE fracture: Lower extremity fracture

As illustrated in Fig. 9, six specific variables were incorporated into the analysis: age, HB, PTP, LE fracture, PDD3, and PDDR3. These variables were identified as the crossover variables of GLM, LSM, and RFM. Consequently, these crossover variables appear to be significant predictors of VTE and are integral to the predictive models. The variables were weighted in descending order as follows: PDDR3, PDD3, age, HB, LE fracture, and PTP, underscoring the substantial predictive value of PDDR3 for VTE across all models.

### Comparative analysis of the effectiveness of different models

**Fig. 10:**
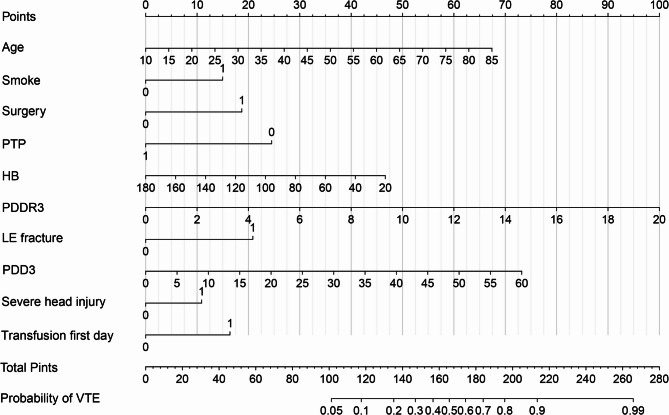
The nomogram of LASSO Model (LSM). PDD1: Post-injury D-dimer on the first day; PDD3: Post-injury D-dimer on the third day; PDDR3: Post-injury D-dimer decrease rate on the third day; HB: Hemoglobin; PTP: Pharmacological thromboprophylaxis; LE fracture: Lower extremity fracture; VTE: Venous thromboembolism

Table 3 presents the performance metrics of the three PDDR3-based models across different cohorts. In the overall cohort, the accuracies for GLM, LSM, and RFM were 68.2%, 72.2%, and 73.4%, respectively. Within the training cohort, the accuracies were 71.0%, 72.4%, and 74.2% for GLM, LSM, and RFM, respectively. In the validation cohort, the accuracies were 73.9%, 80.5%, and 78.7% for GLM, LSM, and RFM, respectively. When considering additional model metrics such as the AUC, sensitivity, specificity, positive predictive value, negative predictive value, and kappa value, the LSM demonstrated superior performance compared to the GLM, while the RFM showed slightly lower predictive efficacy than the GLM. Figure 10 depicts the nomogram of the LSM model, with a total of 10 candidate variables recruited.

**Table 3:** The testing performance of three prediction models in different patient cohorts

Items	AUC (95%CI)	Sensitivity(95%CI)	Specificity(95%CI)	PPV (95%CI)	NPV (95%CI)	Accuracy (%)	Kappa
Overall cohort							
GLM	0.848(0.821–0.875)	0.929(0.891–0.966)	0.634(0.603–0.665)	0.333(0.292–0.374)	0.978(0.967–0.99)	68.2	0.327
LSM	0.849(0.821–0.876)	0.835(0.781–0.889)	0.7(0.67–0.729)	0.353(0.308–0.399)	0.956(0.94–0.971)	72.2	0.346
RFM	0.809(0.777–0.84)	0.786(0.726–0.845)	0.724(0.695–0.752)	0.358(0.311–0.405)	0.945(0.928–0.962)	73.4	0.344
Training cohort							
GLM	0.837(0.804–0.871)	0.877(0.82–0.933)	0.676(0.64–0.712)	0.353(0.301–0.405)	0.965(0.948–0.982)	71.0	0.347
LSM	0.851(0.819–0.883)	0.9(0.848–0.952)	0.688(0.653–0.724)	0.368(0.315–0.421)	0.972(0.956–0.987)	72.4	0.373
RFM	0.82(0.783–0.856)	0.815(0.749–0.882)	0.727(0.693–0.762)	0.376(0.319–0.432)	0.951(0.932–0.97)	74.2	0.37
Validation cohort							
GLM	0.847(0.792–0.902)	0.885(0.798–0.971)	0.712(0.659–0.765)	0.362(0.279–0.446)	0.971(0.948–0.994)	73.9	0.376
LSM	0.853(0.793–0.909)	0.788(0.677–0.899)	0.808(0.762–0.854)	0.432(0.332–0.531)	0.954(0.927–0.98)	80.5	0.446
RFM	0.794(0.731–0.858)	0.769(0.655–0.884)	0.79(0.742–0.838)	0.404(0.307–0.501)	0.949(0.92–0.977)	78.7	0.409

*GLM* Generalized linear model, *LSM* Least absolute shrinkage and selection operator model, *RFM* Random forest model, *PPV* Positive predictive value, *NPV* Negative predictive value, *AUC* Area under the receiver operating characteristics curve, *CI* Confidence interval

## Discussion

Venous thromboembolism represents one of the most prevalent preventable causes of in-hospital mortality [20]. It has been documented that 10–30% of patients with VTE succumb within 30 days, with the majority of fatalities attributable to pulmonary embolism, where sudden death is the initial manifestation in 25% of cases [21, 22]. Patients with TBI are considered to be at a heightened risk for VTE due to factors such as prolonged immobilization, central venous catheterization, extended sedation, mechanical ventilation, and the administration of vasoactive medications [23, 24]. In managing VTE for TBI patients, two critical principles must be addressed: firstly, the increased risk of VTE in this demographic necessitates careful attention; secondly, the advantages of PTP must be balanced against the risk of exacerbating intracranial hemorrhage [25, 26]. Consequently, there is an urgent need for reliable methods of risk stratification and early detection of high-risk individuals. In this study, we successfully developed a VTE prediction model based on PDDR3 utilizing machine learning techniques, which will enhance the early identification of high-risk patients for VTE and support personalized clinical management.

In our study, we initially included all patients with TBI admitted to our trauma center. Subsequently, to investigate early predictive models for VTE in TBI patients, we excluded patients who were not admitted on the day of their injury. To mitigate the effects of age distribution, we further excluded the pregnant and underage patients. Additionally, to control for the influence of concurrent medications, we excluded patients with recent coagulation insufficiency or those using anticoagulant or antiplatelet therapies. Patients with a history of malignant neoplastic disease or incomplete clinical data were also excluded. As for predictive models, three machine learning techniques, including GLM, LSM, and RFM were employed to identify variables significantly influencing VTE. The GLM is an extension of traditional linear models, which is renowned for its interpretability and has been extensively utilized in the construction of predictive models [27]. With the rapid advancements in artificial intelligence, novel predictive models based on machine and deep learning have demonstrated superior accuracy compared to traditional methods, thereby questioning the efficacy of the traditional mothed [28]. Our findings indicate that the AUROC and sensitivity of the LASSO method slightly surpasses that of the GLM, whereas the performance of the RF method is marginally inferior to the GLM. These results suggest that while the evolution of artificial intelligence techniques has introduced numerous innovative methodologies, traditional methods can also demonstrate excellent stability with the help of deep learning. The judicious and appropriate application of deep learning methods can facilitate the development of more stable, efficient, and practical clinical tools.

Interestingly, the three PDDR3-based models incorporate six cross-sectional factors: age, HB, PTP, LE fracture, PDD3 and PDDR3. These factors are pivotal in understanding the increased incidence of VTE in patients with TBI. Previous research has identified age, LE fracture, and PTP as significant contributors to VTE risk in TBI patients [29, 30]. Elderly individuals are often affected by chronic conditions such as diabetes, cardiac disorders, and varicose veins in the lower extremities. Studies indicate that older patients experience reduced vascular elasticity, diminished blood flow, and a heightened propensity for venous stasis following injury, all of which facilitate VTE development [31]. Lower extremity fractures frequently lead to VTE due to endothelial damage, extended periods of immobility, and multiple surgical interventions. PTP treatment can mitigate the hypercoagulable state and decelerate platelet aggregation and thrombosis. However, the improper administration of anticoagulants and antiplatelet agents may elevate the risk of coagulation abnormalities and subsequent hemorrhagic complications.

The plasma D-dimer assay, which is derived from cross-linked fibrinolytic products resulting from the degradation of the fibrin matrix of fresh venous thrombi, is a safe and cost-effective method for assessing VTE. The absolute level of D-dimer correlates positively with thrombus load and is significantly elevated in patients with proximal DVT compared to those with distal DVT. Consequently, the plasma D-dimer assay may be beneficial in distinguishing patients with a high thrombotic load [32, 33]. Studies have demonstrated that the sensitivity and negative predictive value of the D-dimer assay for excluding VTE are 95.2% and 96.2%, respectively. However, the specificity and positive predictive value are relatively low, at 55.3% and 48.7%, respectively [9, 32–34]. Therefore, D-dimer assay is frequently employed as a primary screening tool for VTE. Nevertheless, D-dimer levels in trauma patients typically exhibit a rapid increase followed by a gradual return to baseline, significantly limiting their predictive utility in individuals with TBI [33, 35]. Previous studies have indicated that plasma D-dimer levels in the majority of trauma patients normalize within 3–7 days post-injury [36, 37]. Notably, our study introduced the use of PDDR3 to capture the dynamic fluctuations in plasma D-dimer levels, an aspect not previously explored. These dynamic assessments may mitigate the limitations posed by the low specificity of the D-dimer assay. We posited that patients exhibiting a slower reduction in D-dimer levels might remain in a prolonged hypercoagulable state, thereby elevating their risk of VTE. Our results substantiate the significant prognostic value of PDD3 and PDDR3 in the early prediction of VTE following TBI.

It has been reported that approximately one in five patients with moderate to severe TBI may develop deep vein thrombosis or pulmonary embolism [30, 38]. Additionally, severe injury, advanced age, and obesity are significant risk factors for VTE in TBI patients. Therefore, it is essential to implement a more aggressive surveillance program with effective protective measures for this population as early as possible [39–41]. Furthermore, some researchers have found that nearly one-third of VTE events occur within three days after injury [42, 43]indicating that the monitoring for VTE in TBI patients should commence as soon as they are admitted. In our study, the PDDR3-based model effectively identifies high-risk individuals for VTE within three days following TBI, which may facilitate personalized thromboprophylaxis treatment.

Currently, there is no uniform standard prophylactic strategy for PTP treatment after TBI. Some guidelines indicate that a delay of 48 to 72 h in initiating anticoagulation therapy after the confirmed stabilization of intracranial hemorrhage does not increase the risk of secondary hemorrhage [39, 44]. However, a common delay or even the absence of anticoagulation therapy persists due to limitations in CT imaging technology, which can hinder timely access to head CT scans for some patients. In recent years, an increasing number of scholars have advocated that early PTP within 72 h after injury can reduce the incidence of VTE without raising the rates of secondary intracranial hemorrhage and mortality [42, 45]. Nevertheless, routine early PTP therapy should be approached with caution to avoid overtreatment. Encouragingly, our results demonstrated that the PDDR3-based clinical prediction model achieved an AUROC value of 0.85, combined with the nomogram scoring system of LSM, we were able to effectively distinguishing high-risk patients for VTE at an early stage. This capability can assist clinicians in delivering timely and personalized preventive treatment for each patient.

## Conclusions

In summary, PDD3 and PDDR3 serve as effective biomarkers for the early prediction of VTE following TBI. Through the application of a machine learning algorithm, we have successfully developed a practical clinical model based on PDDR3. This model facilitates the efficient and timely identification of high-risk individuals, thereby enhancing clinical decision-making and treatment strategies.

## Data Availability

The datasets supporting the conclusions of this article are included within the article and its supplementary files. All materials used in this manuscript will be made available to researchers, subject to confidentiality agreements.
